# Feasibility and responsiveness to an electronic system to collect long-term patient-reported outcome measures after rectal cancer resection

**DOI:** 10.1093/bjsopen/zraf053

**Published:** 2025-05-17

**Authors:** Annalisa Maroli, Filippo Bianchi, Caterina Foppa, Stefano De Zanet, Federico Zangrandi, Michele Carvello, Carlotta La Raja, Antonino Spinelli

**Affiliations:** IRCCS Humanitas Research Hospital, Division of Colon and Rectal Surgery, Milan, Italy; IRCCS Humanitas Research Hospital, Division of Colon and Rectal Surgery, Milan, Italy; IRCCS Humanitas Research Hospital, Division of Colon and Rectal Surgery, Milan, Italy; Humanitas University, Department of Biomedical Sciences, Milan, Italy; IRCCS Humanitas Research Hospital, Division of Colon and Rectal Surgery, Milan, Italy; IRCCS Humanitas Research Hospital, General Direction Department, Milan, Italy; IRCCS Humanitas Research Hospital, Division of Colon and Rectal Surgery, Milan, Italy; Humanitas University, Department of Biomedical Sciences, Milan, Italy; IRCCS Humanitas Research Hospital, Division of Colon and Rectal Surgery, Milan, Italy; Humanitas University, Department of Biomedical Sciences, Milan, Italy; IRCCS Humanitas Research Hospital, Division of Colon and Rectal Surgery, Milan, Italy; Humanitas University, Department of Biomedical Sciences, Milan, Italy

Patient-reported outcome measures (PROMs) are increasingly recognized as key metrics of surgical quality, including functional outcomes^1^. The use of electronic PROMs (ePROMs) has several theoretical advantages, but may be limited by both hospital-related and patient-related factors^2^.

This study explored the feasibility and responsiveness of ePROMs after rectal cancer surgery in a retrospective cohort of 245 patients who underwent total mesorectal excision in a tertiary referral centre between January 2015 and January 2022. The electronic survey included the low anterior resection syndrome (LARS) score^3^ and was sent to patients through short message service (SMS) and email. Before sending out the survey to all patients, a sample of 34 patients, randomly selected using statistical software, received a telephone call from the research team before being sent the survey. Patients who did not complete the survey were contacted, including during outpatient appointments, to determine why they had not returned the survey and, when possible, to get them to complete the LARS score. Using this strategy, the LARS score was collected from 27 non-responders.

After survey delivery to the entire cohort, 125 patients (51.0%) completed the survey in a median of 4.00 (interquartile range (i.q.r.) 2.40–6.19) min. Three patients (1.2%) experienced a technical issue preventing the survey completion. Patients who did not complete the survey were telephoned; the most common reason for not completing the survey (83 patients, 33.8%) was a lack of time (*Table S1*). Comparative analysis showed that a significantly higher number of patients who completed the survey had been involved in previous research, were prealerted by a telephone call from the research team, and had major LARS symptoms than those who did not complete the survey (*Table S2*). Multivariable logistic regression analysis confirmed that having major LARS symptoms was an independent factor associated with an ePROMs response (*Table 1*).

**Table 1:** Multivariable analysis of the factors influencing survey responsiveness

	OR	*P*
Previous research involvement	1.01 (0.39, 2.54)	0.995
Survey pre-alert	2.66 (0.56, 12.66)	0.219
Major LARS	5.51 (1.95, 15.57)	0.001

Values in parentheses are 95% confidence intervals. The logistic regression model was statistically significant (Omnibus test: $\chi_{3}^{2}$ = 15.19; *P =* 0.002). The model explained 16% (Nagelkerke *R*^2^) of the variance of survey responsiveness and correctly classified 82% of cases. The Hosmer-Lemeshow goodness-of-fit test of the final model ($\chi_{4}^{2}$ = 1.003; *P* = 0.909) indicated adequate fitness. OR, odds ratio; LARS, low anterior resection syndrome.

This study demonstrated that survey (ePROMs) delivery and collection were feasible and could result in adequate response rates among patients who had undergone rectal surgery. Survey responsiveness was increased by a prealert from research staff, confirming the pivotal role of the healthcare providers in patient engagement. Another factor contributing to the survey responsiveness, as confirmed in multivariable analysis, was the presence of major LARS symptoms: non-responders had a significantly lower LARS scores than responders (median 13 (i.q.r. 4–26) *versus* 32 (27–37); *P* < 0.0001) (*Fig. S1*). This suggests that, as noted in previous studies^4^, the perceived relevance of the survey may significantly impact patient feedback. However, the LARS score was retrieved from only 27 non-responders and the multivariable model explained only 16% of the variance, suggesting that other factors may contribute to survey responsiveness and that larger, prospective studies are needed to confirm these findings. These results suggest that, although providing direct and unbiased feedback from patients, the use of ePROMs may introduce selection bias. Although ePROMS are a valid clinical tool allowing for the prompt identification of patients with severe symptoms, they should be interpreted with caution when it comes to collecting prevalence data.

In conclusion, this study demonstrated that ePROMs are technically feasible and can provide high response rates when introduced by hospital staff. Symptoms severity may represent a main driver for responsiveness, making ePROMs a useful tool for promptly identifying functional impairments.

## Supplementary Material

zraf053_Supplementary_Data

## Data Availability

The study data will be uploaded in the public repository Zenodo (https://zenodo.org/) according to local standard operating procedures with an appropriate embargo. Pseudo-anonymized data will be made available upon reasonable request.
