# Supplementary material for: Feasibility and responsiveness to an electronic system to collect long-term patient-reported outcome measures after rectal cancer resection
Source: BJS Open. 2025 May 17;9(3):zraf053. doi: 10.1093/bjsopen/zraf053 (PMC12084676; doi:10.1093/bjsopen/zraf053)
Supplement: zraf053_Supplementary_Data [file zraf053_supplementary_data.zip › Supplementary_material.docx]

**Research letter**

**Feasibility and responsiveness to an electronic system to collect long-term Patients Reported Outcomes Measures (PROMs) after rectal cancer resection**

Annalisa Maroli^2^, Filippo Bianchi^2^, Caterina Foppa^1,2^, Stefano De Zanet^2^, Federico Zangrandi^2^, Michele Carvello^1,2^, Carlotta La Raja^1,2^, Antonino Spinelli^1,2^

^1^ Department of Biomedical Sciences, Humanitas University, via Montalcini 4, Pieve Emanuele (MI), 20090, Italy

^2^ IRCCS Humanitas Research Hospital, via Manzoni 56, Rozzano (MI), 20089, Italy

Corresponding author:

Prof. Antonino Spinelli, M.D., Ph.D.

Director, Division of Colon and Rectal Surgery

IRCCS Humanitas Research Hospital

Via Manzoni 56, Rozzano (MI), 20089, Italy

[Antonino.spinelli@hunimed.eu](mailto:Antonino.spinelli@hunimed.eu)

+39 02 82247776

ORCID ID: 0000-0002-1493-1768

**Supplementary Materials - Index**

| **Supplementary Figures and Tables** |  |
| --- | --- |
| Table S1 | *Page 2* |
| Table S2 | *Page 3* |
| Figure S1 | *Page 4* |
| Figure S2 | *Page 5* |

**Supplementary Figures and Tables**

| **Table S1. Survey responsiveness** | |
| --- | --- |
| Survey completeness |  |
| Not opened | 87 (35.5%) |
| Death | 4 (4.5%) |
| Technical issue | 3 (3.4%) |
| Lack of time/desire | 25 (28.7%) |
| Critical illness/disabilities | 5 (5.7%) |
| Caregiver’s reference number | 11 (12.6%) |
| Language barrier | 2 (2.2%) |
| Lost to follow-up | 37 (42.5%) |
| Opened but not started | 30 (12.2%) |
| Lack of time/ desire | 14 (46.6%) |
| Critical illness/disabilities | 5 (16.6%) |
| Caregiver’s reference number | 7 (23.3%) |
| Language barrier | 1 (0.3%) |
| Stoma replacement | 3 (10%) |
| Started but not completed | 3 (1.2%) |
| Completed | 125 (51%) |
| Time from survey reception and opening (minutes)* | 89 [12-384] |
| Time for survey completion (minutes)* | 4.00 [2.40-6.19] |
| Number of openings* | 2 [1-4] |
| Type of device† |  |
| Smartphone | 137 (86.7%) |
| Tablet | 1 (0.6%) |
| Personal computer | 20 (12.6%) |
| Abbreviations: IQR, Interquartile Range.  Values in parentheses are percentages unless indicated otherwise; values are *median [interquartile range].  †Percentage calculated over patients who opened the survey (n= 158) | |

| **Table S2. Demographic and clinical characteristics of responder and non-responder patients** | | | |
| --- | --- | --- | --- |
|  | **Responders** | **Non-responders** | ***p*-value** |
| Number of patients | 125 | 120 |  |
| Age (years)* | 66.88 ± 10.51 | 66.23 ± 13.29 |  |
| Gender, females | 49 (39.2%) | 48 (40%) | 1.000 |
| Domicile distance from the centre (Km)† | 64.60 [34.45-833.00] | 80.00 [37.53-802.30] | 0.564 |
| Intra-region domicile | 67 (53.6%) | 58 (48.3%) | 0.444 |
| Followed by the investigational centre | 68 (72.4%) | 64 (53.8%) | 0.172 |
| Previous research involvement | 78 (62.4%) | 50 (41.7%) | 0.001 |
| Survey pre-alert | 23 (18.4%) | 11 (9.2%) | 0.043 |
| Working status |  |  | 0.152 |
| Employed | 54 (43.2%) | 41 (34.2%) |  |
| Unemployed | 71 (56.8%) | 79 (65.6%) |  |
| Education level |  |  | 0.608 |
| High degree | 73 (58.4%) | 66 (55%) |  |
| Low degree | 52 (41.6%) | 54 (45%) |  |
| Time from surgery (or stoma closure) to survey compilation (days)† | 1387 [916-1979] | 1154 [785-1838] | 0.089 |
| Stoma construction | 118 (94.4%) | 110 (91.7%) | 0.457 |
| Complications after rectal resection | 57 (45.6%) | 46 (38.3%) | 0.300 |
| Complications after stoma closure | 2 (1.7%) | 6 (5.4%) | 0.160 |
| LARS‡ | 108 (86.4%) | 10 (37%) | <0.0001 |
| Major LARS‡ | 70 (56%) | 5 (18.5%) | 0.001 |
| Abbreviations: IQR, Interquartile Range; LARS, Low Anterior Resection Syndrome.  Values in parentheses are percentages unless indicated otherwise; values are *mean ± standard deviation and †median [interquartile range].  ‡Percentage calculated over 125 responders and 27 non-responders | | | |

**Figure S1:** Low Anterior Resection Syndrome (LARS) scores in responders (black box) and non-responder patients (red box). The LARS score of non-responders was significantly lower compared with responders (13 [4-26] vs 32 [27-37]; *p*< 0.0001).

**Figure S2:** Visual abstract of the study.
