# Supplementary figures and images for: Feasibility and responsiveness to an electronic system to collect long-term patient-reported outcome measures after rectal cancer resection
Source: BJS Open. 2025 May 17;9(3):zraf053. doi: 10.1093/bjsopen/zraf053 (PMC12084676; doi:10.1093/bjsopen/zraf053)

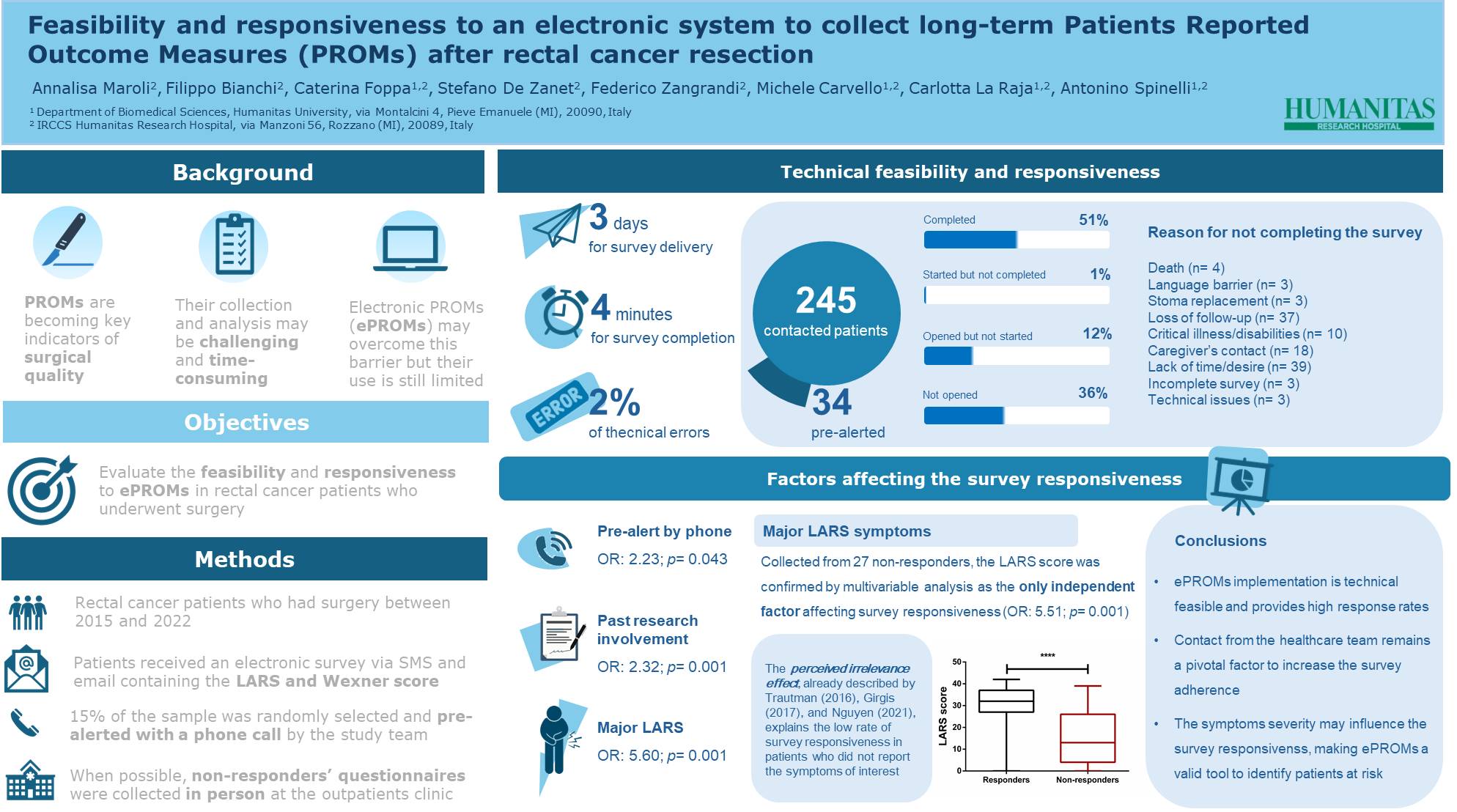

Supplement: zraf053_Supplementary_Data [file zraf053_supplementary_data.zip › Figure_S2.jpg]
